# Supplementary material for: Survival and Death Causes in Thyroid Cancer in Taiwan: A Nationwide Case–Control Cohort Study
Source: Cancers (Basel). 2021 Aug 5;13(16):3955. doi: 10.3390/cancers13163955 (PMC8391882; doi:10.3390/cancers13163955)
Supplement: Supplementary file 1 [file cancers-13-03955-s001.zip › cancers-1283772-supplementary.pdf]

Table S1: ICD codes of diseases.

Disease	ICD-9	ICD-10
Thyroid cancer	193	C73
Ischemic heart disease	410, 411.1, 412, 413, 414, 429.5, 429.6, 429.71, 429.79, 429.2	I20-I25
Ischemic stroke	433.01, 433.11, 433.21, 433.31, 433.81, 433.91, 434.01, 434.11, 434.91	I63
Hemorrhagic stroke	430, 431, 432	I60-I62, I64
Hyperlipidemia	272	E78
Diabetes mellitus	250	E10.0, E10.1, E10.9-E11.1, E11.9
Hypertension	401, 402	I10-I16
Malignancies other than thyroid cancer	140-208 (except 193)	C00-C97 (except C73)
Cardiovascular disease	390-398, 410-414, 420-429, 401-405, 430-438, 440	I10-I15, I01-I02. 0, I05-I09, I20-I25, I27, I30-I52, I60-I71
Diabetes mellitus	250	E10-E14
Infectious disease	001-018, 033, 036-038, 050, 055, 084, 320-322, 480-487	A00-A09, A15 -A19, A40-A41, G00, G03, J10-J18, B15-B24
Injury/trauma	800-969	V00-X59, Y85-Y86, X60-X84, Y87.0, X85-Y09, Y87.1
Renal disease	580-589	N00-N07, N17-N19, N25-N27
Digestive disease	531-533, 540-543, 571	K25-K28, K40-K46, K56, K70, K73-K74, K80-K82
Lower respiratory disease	490-493	J20-J21, J40-J47, J60-J65
Anemia, malnutrition and age-related debility	260, 262-263, 280-285, 600, 630-646, 651-676, 760-779	D50-D64, O00-O99, P00-P96, G12, R54, G20-G21, G30, L00-L99, M00-M99, J66, J68-J69, F01-F03
